# The prevalence and risk factors for phantom limb pain in people with amputations: A systematic review and meta-analysis

**DOI:** 10.1371/journal.pone.0240431

**Published:** 2020-10-14

**Authors:** Katleho Limakatso, Gillian J. Bedwell, Victoria J. Madden, Romy Parker

**Affiliations:** 1 Department of Anaesthesia and Perioperative Medicine, Pain Management Unit, Neuroscience Institute, University of Cape Town, Cape Town, South Africa; 2 Division of Physiotherapy, Department of Health and Rehabilitation Sciences, University of Cape Town, Cape Town, South Africa; West Park Healthcare Centre, CANADA

## Abstract

**Background:**

Phantom limb pain (PLP)—pain felt in the amputated limb–is often accompanied by significant suffering. Estimates of the burden of PLP have provided conflicting data. To obtain a robust estimate of the burden of PLP, we gathered and critically appraised the literature on the prevalence and risk factors associated with PLP in people with limb amputations.

**Methods:**

Articles published between 1980 and July 2019 were identified through a systematic search of the following electronic databases: MEDLINE/PubMed, PsycINFO, PsycArticles, Cumulative Index to Nursing and Allied Health Literature, Africa-Wide Information, Health Source: Nursing/Academic Edition, SCOPUS, Web of Science and Academic Search Premier. Grey literature was searched on databases for preprints. Two reviewers independently conducted the screening of articles, data extraction and risk of bias assessment. The meta-analyses were conducted using the random effects model. A statistically significant level for the analyses was set at p<0.05.

**Results:**

The pooling of all studies demonstrated a prevalence estimate of 64% [95% CI: 60.01–68.05] with high heterogeneity [I^2^ = 95.95% (95% CI: 95.10–96.60)]. The prevalence of PLP was significantly lower in developing countries compared to developed countries [53.98% vs 66.55%; p = 0.03]. Persistent pre-operative pain, proximal site of amputation, stump pain, lower limb amputation and phantom sensations were identified as risk factors for PLP.

**Conclusion:**

This systematic review and meta-analysis estimates that six of every 10 people with an amputation report PLP–a high and important prevalence of PLP. Healthcare professionals ought to be aware of the high rates of PLP and implement strategies to reduce PLP by addressing known risk factors, specifically those identified by the current study.

## Introduction

Phantom limb pain (PLP)—pain felt in the amputated limb–is often accompanied by significant suffering [1]. The condition is difficult to manage and can lead to disability and reduced health-related quality of life [2]. Several risk factors, including stump pain, diabetic cause of amputation and depression, have been found to be associated with the onset and continuation of PLP [3–5]. While there are reports of PLP in people with congenital amputations [6], PLP appears to be more prevalent in people with traumatic or surgical limb amputations [7].

There are conflicting reports on the prevalence of PLP in people with limb amputations. Whereas one study reported a high prevalence of 85.6% [8], another reported a substantially lower prevalence of 29% [9]. The inconsistent reports on the prevalence of PLP are perplexing, but may be due to differences in the study samples (upper- vs lower-limb amputees or mixed populations), countries in which the research was undertaken, and methodologies between studies [10].

Higher prevalence rates of PLP have been reported in people with lower limb amputations than in those with upper limb amputations [4, 11]. Lower limb(s) amputations are performed chiefly to treat complications of diabetes, and may be associated with risk factors for PLP such as pre-amputation pain and depression [12]. The high PLP prevalence could be explained by these risk factors, which are typically absent in people with upper limb amputations, who are typically healthy and undergo amputation due to trauma [13]. Studies that report PLP prevalence in people receiving continuing medical care have a selection bias [3, 4, 14], in that they fail to account for patients not receiving continuing medical care, who may have different prevalence rates [15]. In addition, previous studies suggest that PLP prevalence rates may be lower in developing countries [16]. However, no clear hypothesis for these lower prevalence rates has been proposed. Further, it is not clear if the prevalence rates in developing countries are significantly lower to those seen in developed countries.

Epidemiological studies are essential to inform health care professionals and health system planners about the burden of diseases in a population [17]. Estimates of the burden of PLP have provided conflicting data. To our knowledge, no systematic review has been conducted on the prevalence and risk factors for PLP. Therefore, to obtain an accurate estimate of the burden of PLP, we gathered and critically appraised the literature on the prevalence and risk factors associated with PLP in people with limb amputations.

The primary aim of this systematic review was to estimate the prevalence of PLP in people with limb amputations. The secondary aim was to determine whether there is a difference in the prevalence of PLP in developed and developing countries, as per the World Economic Situation and Prospects classification system [18]. The exploratory aim was to identify risk factors associated with PLP in people with limb amputations.

## Materials and methods

This systematic review was designed according to the Preferred Reporting Items of Systematic Reviews and Meta-Analysis (PRISMA) guidelines [19]. The review protocol was registered on PROSPERO [ID: CRD42018094821], and published in Systematic Reviews [15]. The PRISMA criteria fulfilled by this systematic review are presented in S1 File.

### Data sources and search procedure

The lead investigator (KL) and a senior librarian (MS) developed a comprehensive search strategy (S2 File) using five Medical Subject Headings (MeSH): prevalence, risk factors, amputation, phantom limb and epidemiology. Articles published between 1980 and July 2019 were identified through a systematic search of the following electronic databases: MEDLINE/PubMed (via EBSCOhost), PsycINFO (via EBSCOhost), PsycArticles, Cumulative Index to Nursing and Allied Health Literature (CINAHL) (via EBSCOhost), Africa-Wide Information (via EBSCOhost), Health Source: Nursing/Academic Edition (via EBSCOhost), SCOPUS, Web of Science and Academic Search Premier (via EBSCOhost). We deviated from protocol and searched for grey literature on bioRxiv (www.biorxiv.org), Preprints (www.preprints.org), Open Science Framework (www.osf.io) and medRxiv (www.medrxiv.org). The reference lists of eligible studies were searched manually to identify more studies that may have been eligible for inclusion in this review. Studies identified from the literature search were saved using the citation manager software programme (EndNote x8), which was also used to remove duplicates [20].

### Study selection

**Fig 1:**
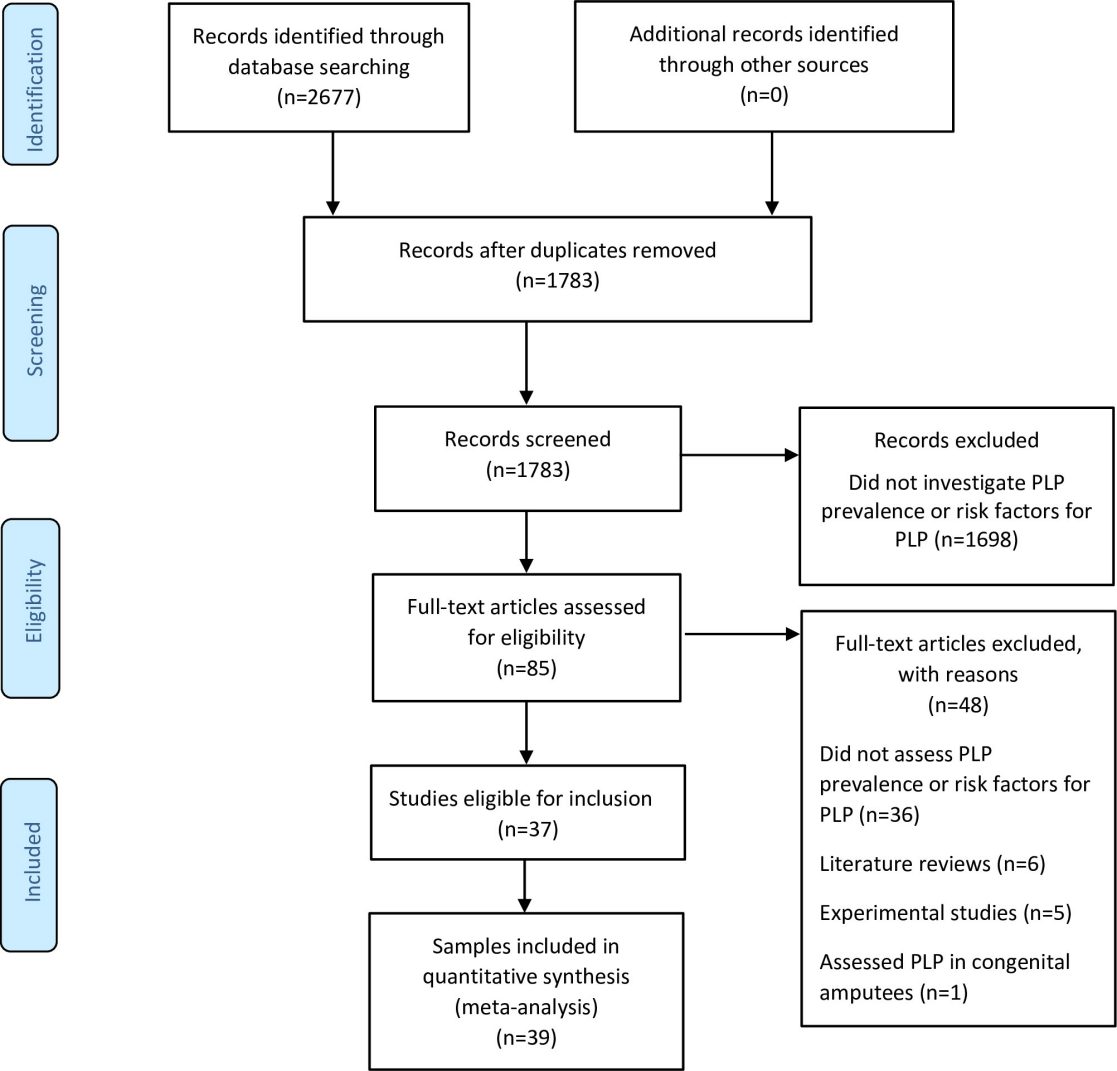
The PRISMA flow diagram illustrating the screening process.

We included cross-sectional, cohort and case-control studies that investigated the prevalence of PLP in adults (18 years or older) with surgical and traumatic upper or lower limb amputations. Only studies written in English, with full text published between 1980 and 2019, were eligible for inclusion in this review. The risk factors for PLP were identified from the included studies. We excluded literature reviews and experimental studies. Two reviewers (KL and GJB) independently screened study titles and abstracts for eligibility. Studies identified in the initial screening as potentially eligible were assessed for eligibility in full-text form by the same reviewers, using the inclusion/exclusion criteria. The study selection procedure was performed using a Microsoft Excel spreadsheet (2016) on which the studies were listed and marked as either eligible or ineligible. In this, we deviated from the registered protocol, which specified the use of Covidence, because Covidence has limited usability offline. A PRISMA flow diagram (Fig 1) represents the entire screening process detailing the numbers of included and excluded studies, with reasons for exclusion. After each stage, results were compared, and disagreements resolved through discussion.

### Risk of bias assessment

Two reviewers (KL and GJB) independently assessed the included articles for risk of bias using a risk of bias assessment tool for prevalence studies that had been developed by Hoy et al (S3 File) [21]. This tool assesses the risk of bias based on 10 categories which evaluate the study’s external and internal validity. Each category of the risk of bias tool was set as “high risk” if the study scored “high risk” for any single item within that category, and “low risk” if it scored “low risk” for all items in that category. Categories with “low risk” and “high risk” were given a rating of zero and one respectively. The summary risk of bias rating for each study was presented as “low risk” (score: 0–3), “moderate risk” (score: 4–6), or “high risk” (score: 7–10).

### Data extraction

Two reviewers (KL and GJB) used a pre-piloted customised data extraction sheet to independently extract relevant data from included studies. Data extracted included: the names of authors, year of study publication, study design and setting, country of study, sample size, participants’ age and sex, site of amputation, method of data collection, PLP prevalence (%), as well as risk factors and their measures of association with PLP. The developmental status of each country was identified using the World Economic Situation and Prospects classification system [18].

### Data analysis

Data extracted from individual studies were entered into an Excel spreadsheet for analysis. All meta-analyses were conducted using Open Meta Analyst software available on (http://www.cebm.brown.edu/openmeta). In this, we deviated from the registered protocol, which specified the use of Review Manager 5, because Review Manager 5 is not suitable for conducting meta-analyses of single arm studies. Cohen's Kappa was used to report inter-rater agreement during screening, data extraction and risk of bias assessment, and can be interpreted as minimal (0–0.39), weak (0.40–0.59), moderate (0.60–0.79) or strong (0.80–0.90) [22]. Clinical heterogeneity was evaluated qualitatively, based on similarities or differences in participant and outcome characteristics, recruitment and assessment procedures, and study setting [23]. Statistical heterogeneity was assessed using the *I*^*2*^ statistic, and the results were presented as low (<25%), moderate (25–50%) and high (>50%) [24]. Subject to consideration of heterogeneity and risk of bias, studies were pooled for meta-analysis using a random effects model to determine a sample-weighted summary estimate of PLP prevalence. A funnel plot was generated to assess for possible publication bias. Furthermore, the Egger’s test was conducted to assess the funnel plot for asymmetry [25]. To address high statistical heterogeneity, we deviated from protocol to sub-group studies into those with low risk of bias and those with moderate and high risk of bias, and conducted separate meta-analyses, and compared the two pooled estimates using a two-tailed Mann-Whitney U test. We also sub-grouped studies by the developmental status of the country in which each study had been conducted [18], as planned in the protocol and, again, compared the estimates using a two-tailed Mann-Whitney U test. Potential risk factors for PLP were identified from the included studies and analysed descriptively. When an association was confirmed, the strength of association between PLP and each risk factor was classified as either “weak”, “moderate”, “strong” or “very strong”, according to the guidelines for interpreting the strength of association in epidemiology studies [26–29]. We calculated Phi (Ø) to determine the strength of association in studies that used the chi-square test as a measure of association. This sample-size-adjusted chi-square statistic has been shown to provide a more accurate reflection of the strength of association between two variables than the interpretation of chi-square and probability (P) values, where high chi-square and p values are thought to represent a strong association between variables [30]. Alpha was set at 0.05 for all analyses.

## Results

The initial literature search returned 2677 records, of which 1783 remained after the removal of duplicates. Initial screening of titles and abstracts identified 85 studies that were eligible for full-text review. Full-text review identified 37 studies that were eligible for inclusion in this systematic review. Two of these studies reported two studies each [31, 32]. Therefore, a total of 39 data sets were included in our analysis. The entire screening process reflected moderate agreement (Kappa = 0.70) between reviewers.

### Study characteristics

**Fig 2:**
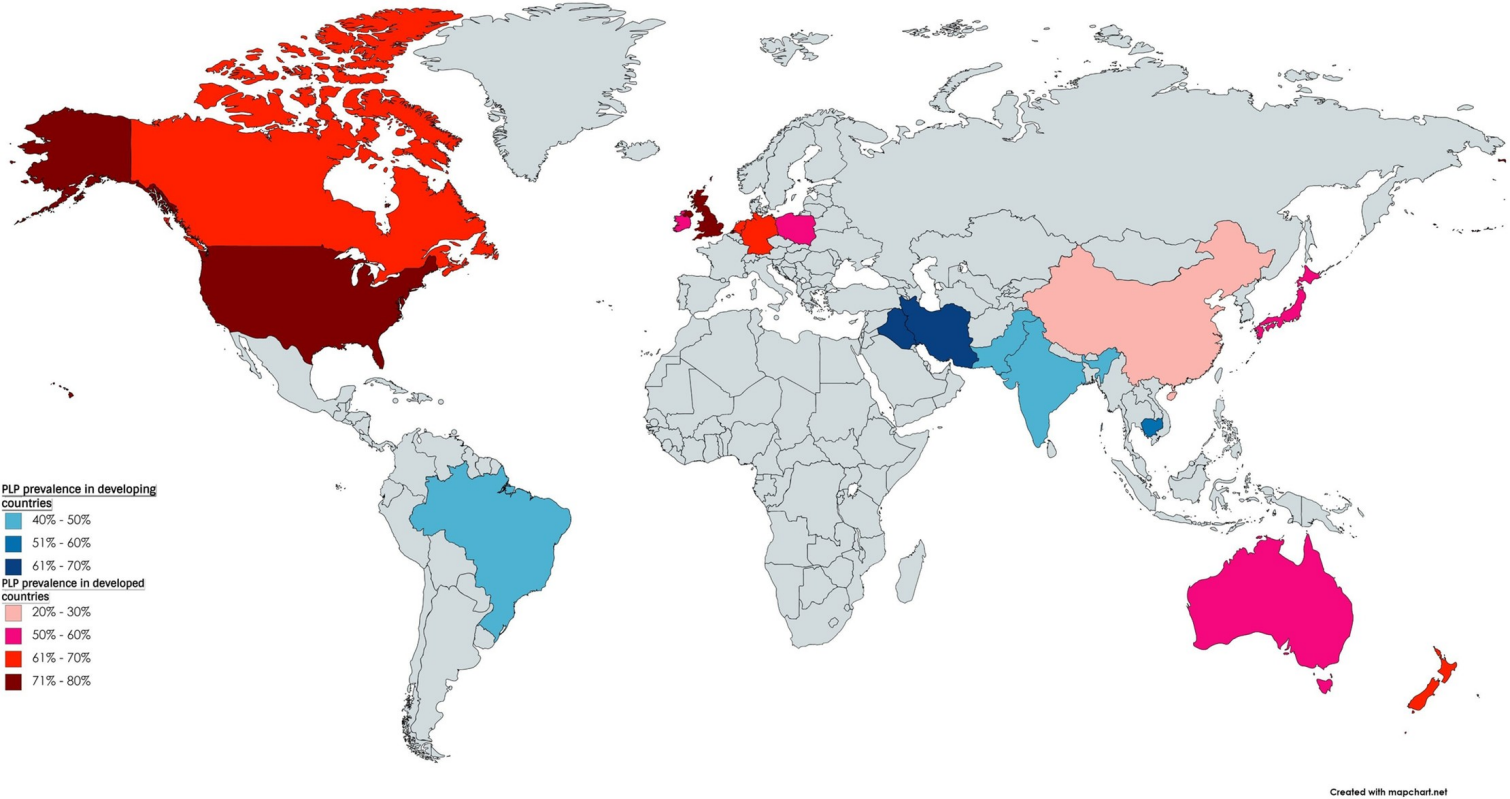
World map showing the countries in which the studies were conducted.

The study characteristics are summarised in Table 1. The included studies had used cross-sectional (n = 35) and cohort (n = 4) study designs. Thirty-two of 39 studies had been conducted in developed countries [18]. Of these, the majority were conducted in North America [USA (n = 7); Canada (n = 2)] and Europe [United Kingdom (n = 8); Germany (n = 5); Netherlands (n = 3); Ireland (n = 2); Poland (n = 1)] (Fig 2). Only seven studies were conducted in developing countries [Iran (n = 2); Iraq (n = 1); India (n = 1); Brazil (n = 1); Pakistan (n = 1); Cambodia (n = 1)]. Table 1 reflects the wide range of data collection approaches used in the studies. The included studies were published between 1986 and 2019. The data extraction process had moderate agreement (Kappa = 0.71) between reviewers prior to discussion.

**Table 1:** Summary of study and participant characteristics by study.

Authors	Study type	Country of study	Development status	Method of data collection	Sample size	Age Mean (SD)	Sex M/F	Level of amputation (UL/LL)	PLP prevalence (%)
Ahmed et al., 2017	Cross-sectional	India	Developing	Self-reported questionnaire	139	38.23 (1.54)	102/37	36/103	41
Aldington et al., 2014	Cross-sectional	UK	Developed	Self-reported questionnaire	48	28.8 (6.7)	-	11/54	49
Bekrater et al., 2015	Cross-sectional	Germany	Developed	Postal and telephone questionnaire	3234	64.37 (15.89)	2637/597	824/2410	62.55
Bin Ayaz et al., 2015	Cross-sectional	Pakistan	Developing	Face-to-face interview	268	28 (6)	266/2	35/233	42.5
Bosmans et al., 2007	Cross-sectional	Netherlands	Developed	Face-to-face interview	16	66.5 (39–86)*	11/5	0/16	81.25
Buchanan et al., 1986	Cross-sectional	Canada	Developed	Face-to-face interview	716	-	616/100	43/647	62.4
Byrne et al., 2011^a^	Cross-sectional	New Zealand	Developed	Face-to-face interview	29	41.7 (4.8)	25/4	7/24	69
Byrne et al., 2011^b^	Cross-sectional	Cambodia	Developing	Face-to-face interview	29	40.3 (10.5)	25/4	1/28	51.7
Clark et al., 2013	Cross-sectional	UK	Developed	Postal and telephone questionnaire	102	70.9 (1.27)	-	0/97	85.6
Datta et al., 2004	Cohort	UK	Developed	Postal questionnaire	60	58.1 (-)	48/12	60/0	60
Desmond et al., 2010	Cross-sectional	Ireland	Developed	Self-reported questionnaire	141	74.8 (-)	138/3	141/0	42.6
Dijkstra et al., 2002	Cross-sectional	Netherlands	Developed	Postal questionnaire	536	-	367/150	99/433	72
Ehde et al., 2000	Cross-sectional	USA	Developed	Postal questionnaire	255	55.1 (14.3)	207/48	0/255	72
Ephraim et al., 2005	Cross-sectional	USA	Developed	telephone interview	914	50.3 (13.3)	552/362	100/812	79.9
Gallagher et al., 2001	Cross-sectional	Ireland	Developed	Postal questionnaire	104	45.3 (18.9)	78/26	0/104	69.2
Hanley et al., 2006	Cross-sectional	USA	Developed	Postal and telephone questionnaire	255	55 (14.3)	207/48	0/255	72
Hanley et al., 2009	Cross-sectional	USA	Developed	Postal questionnaire	104	46.9 (14.1)	75/29	104/0	79
Hnoosh et al., 2014	Cross-sectional	Iraq	Developing	Self-reported questionnaire	118	32 (12.9)	97/21	0/181	61
Houghton et al., 1994	Cross-sectional	UK	Developed	Postal questionnaire	176	71 (-)	-	0/176	78
Kern et al., 2012	Cross-sectional	Germany	Developed	Postal questionnaire	537	59 (-)	382/155	24/513	74.5
Ketz et al., 2008	Cross-sectional	Germany	Developed	Self-reported questionnaire	30	-	30/0	7/27	77
Kooijman et al., 2000	Cross-sectional	Netherlands	Developed	Unclear	72	44.2 (35–65)*	57/15	72/0	51
Larbig et al., 2019	Cohort	Germany	Developed	Face-to-face interview and self-reported questionnaire	52	-	41/11	2/50	75
Morgan et al., 2017	Cross-sectional	USA	Developed	Self-reported and internet questionnaire	1296	54.4 (13.7)	909/387	0/1296	48.1
Noguchi et al., 2019	Cross-sectional	Japan	Developed	Medical records	44	-	33/11	22/22	50
Penna et al., 2018	Cohort	Australia	Developed	Medical records	96	-	74/22	0/96	52.2
Probstner et al., 2010	Cross-sectional	Brazil	Developing	Self-reported questionnaire	75	54.4 (18.5)	50/25	6/69	46.7
Rafferty et al., 2015	Cross-sectional	UK	Developed	Self-reported questionnaire	75	26.3 (18–42)*	74/1	0/84	85
Rahimi et al., 2012	Cross-sectional	Iran	Developing	Face-to-face interview	335	42.1 (6.32)	324/11	0/670	66.7
Rayegani et al., 2010	Cross-sectional	Iran	Developing	Face-to-face interview and self-reported questionnaire	335	-	327/8	0/670	64
Razmus et al., 2017	Cross-sectional	Poland	Developed	Face-to-face interview and self-reported questionnaire	22	61 (11.3)	15/7	3/22	59
Reiber et al., 2010^a^	Cross-sectional	USA	Developed	Postal, internet and telephone questionnaire	298	60.7 (3.0)	298/0	78/300	72.2
Reiber et al., 2010^b^	Cross-sectional	USA	Developed	Postal, internet and telephone questionnaire	283	29.3 (5.8)	274/9	78/273	76
Resnik et al., 2019	Cross-sectional	Canada	Developed	Telephone interview	808	63.2 (14.2)	787/21	840/0	76.1
Richardson et al., 2007	Cohort	UK	Developed	Face-to-Face interview	59	63.8 (10.4)	37/22	0/59	78.8
Richardson et al., 2015	Cross-sectional	UK	Developed	Face-to-face interview	89	65.5 (11.4)	64/25	0/89	63
Schley et al., 2008	Cross-sectional	Germany	Developed	Postal and telephone questionnaire	65	45 (18–80)*	60/5	65/0	44.6
Wartan et al., 1997	Cross-sectional	UK	Developed	unclear	526	73 (-)*	526/0	99/471	62
Yin et al., 2017	Cross-sectional	China	Developed	Telephone interview	391	-	-	-	29

_* Indicates the median age and range._

_The number of amputations and males versus females do not add up to the total sample size because some participants had more than one amputation and these data were not provided._

### Participant characteristics

The included studies provided data from a total of 12738 participants (9814 male; 2183 female) who had undergone upper limb (n = 2757) and lower limb (n = 10539) amputations. Participant characteristics are provided in Table 1.

### Risk of bias assessment

The risk of bias assessment revealed moderate agreement (Kappa = 0.69) between reviewers prior to discussion. The results of the risk of bias assessment are reported in S4 File. Four studies had an overall rating of “low risk” [33–36]. Six studies scored “low risk” for selection bias, for using a sample that was a close representation of the national population [2, 34, 35, 37–39]. Eight studies scored “low risk” for study participation bias, because their response rates for participation were ≥75% [2, 13, 34, 36, 40–43]. Twelve studies scored “low risk” for measurement bias, for using a clear definition of PLP [1, 4, 5, 12, 16, 33, 35–38, 44, 45]. Other studies scored “high risk” for measurement bias, for not providing a clear definition of PLP (e.g. pain felt in the limb after amputation). All the studies scored “high risk” for measurement bias, for using an instrument that has not been shown to be valid and reliable for measuring the outcome of interest. However, all studies scored “low risk” for reporting bias, for appropriately reporting the numerators and denominators for the outcome of interest.

### Prevalence of phantom limb pain

**Fig 3:**
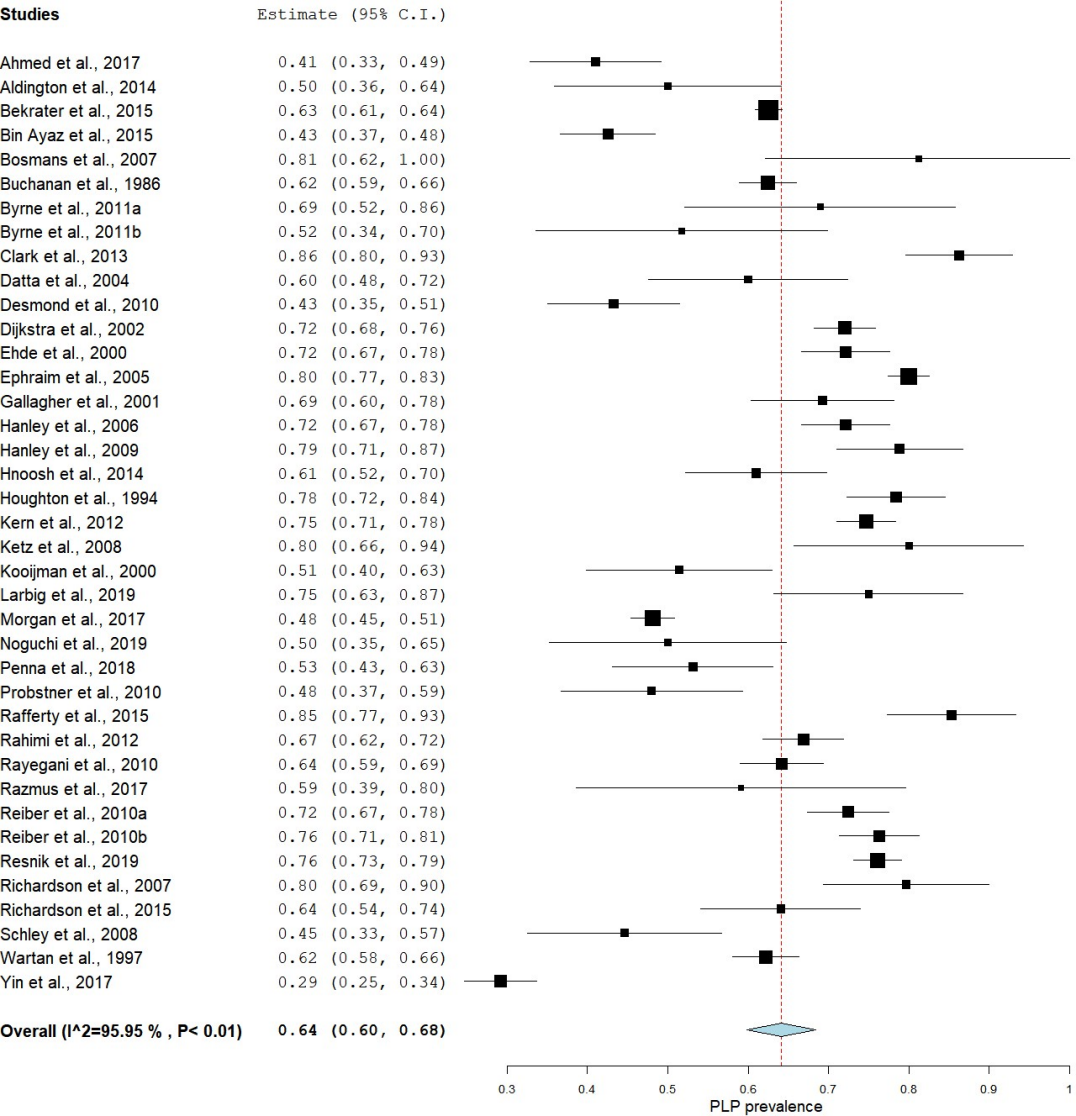
A forest plot showing the overall pooled estimated prevalence of PLP in people with amputations.

The estimates of PLP prevalence in people with limb amputations ranged between 27% and 85.6% [8, 13], with most studies (31 out of 39) reporting a prevalence between 50% and 85.6% [8, 31]. The pooling of all studies using a random effects model yielded an estimated prevalence of 64% [95% CI: 60.01–68.05], but with high statistical heterogeneity [I^2^ = 95.95% (95% CI: 95.10–96.60)] (Fig 3). The Egger’s regression analysis of all the included studies revealed no publication bias [-0.80 (95%CI: -4.32–2.01); p = 0.64].

### Exploratory subgrouping

**Fig 4:**
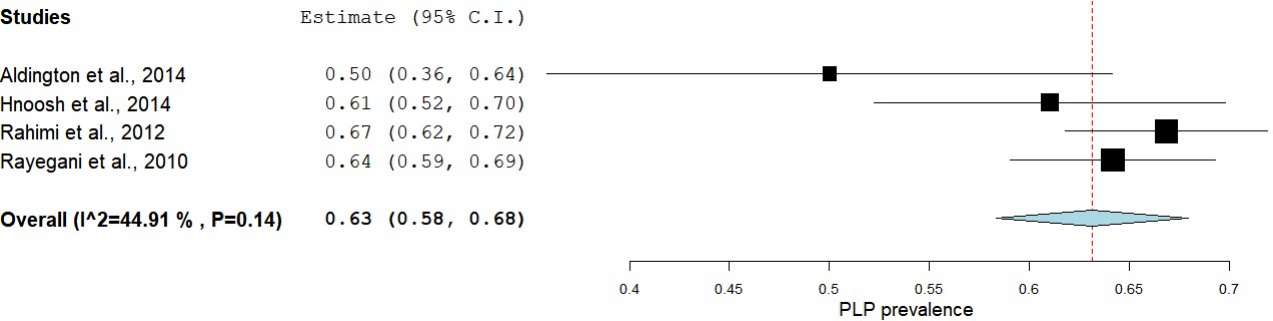
A subgroup analysis showing the pooled estimated prevalence of PLP in studies with low risk of bias.

**Fig 5:**
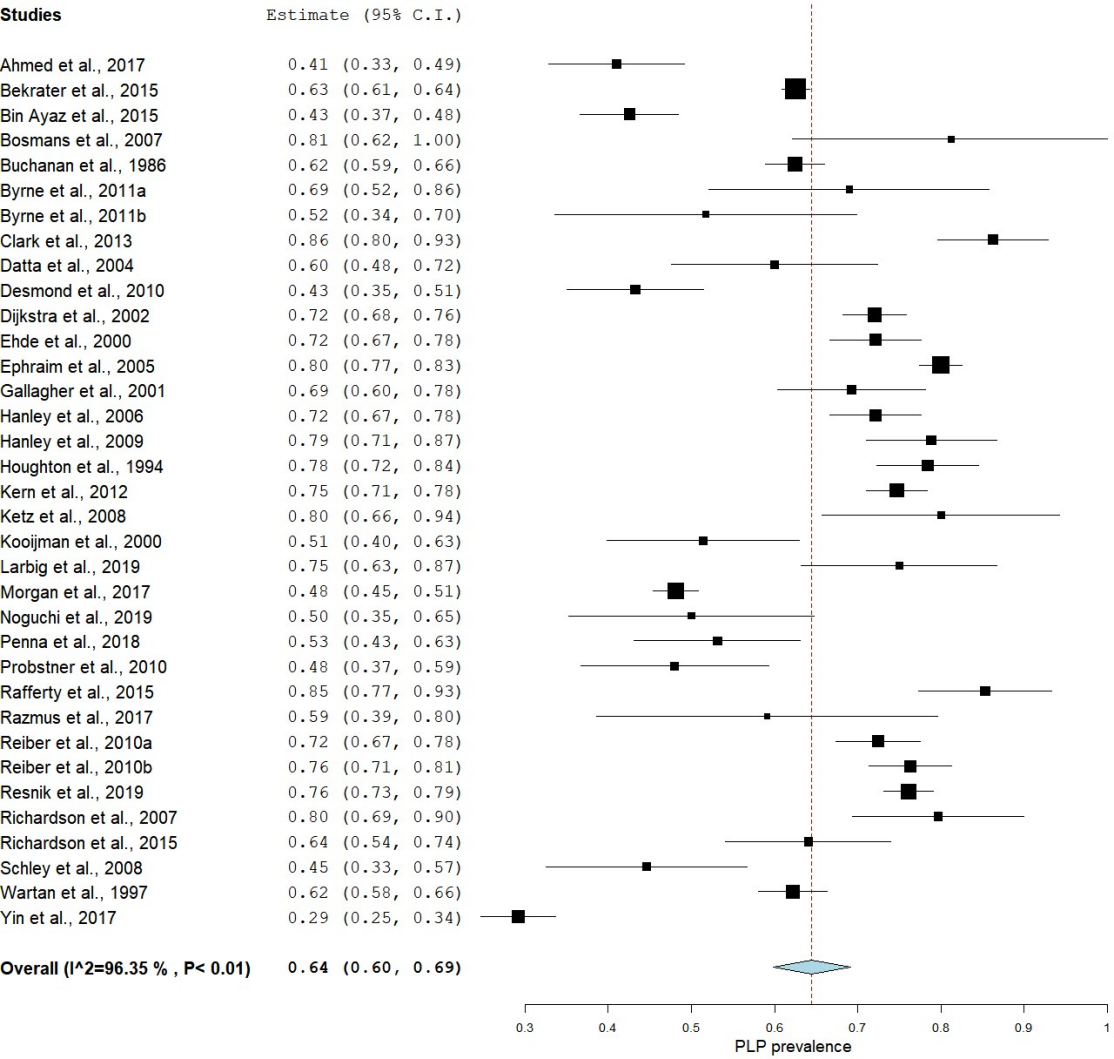
A subgroup analysis showing the pooled estimated prevalence of PLP in studies with moderate to high risk of bias.

We were concerned by the high statistical heterogeneity in the primary meta-analysis, so we opted to deviate from protocol to conduct two exploratory meta-analyses with studies sub-grouped according to risk of bias score. The first exploratory subgroup analysis, including only the studies that scored low risk of bias overall, estimated prevalence at 63% [95% CI: 58.31–67.90] with moderate statistical heterogeneity [I^2^ = 44.91 (95% CI: 43.90–45.20)] (Fig 4). The second exploratory subgroup analysis, including only the studies with moderate-high risk of bias, estimated prevalence at 64% [95% CI: 60.23–69.40], but with high statistical heterogeneity [I^2^ = 96.35% (95% CI: 96.11–98.36)] (Fig 5). The Mann-Whitney U test that served as the sensitivity analysis for the effect of moderate-high risk of bias showed no difference between the estimated prevalence from these two meta-analyses [U = 58.5, p = 0.28].

**Fig 6:**
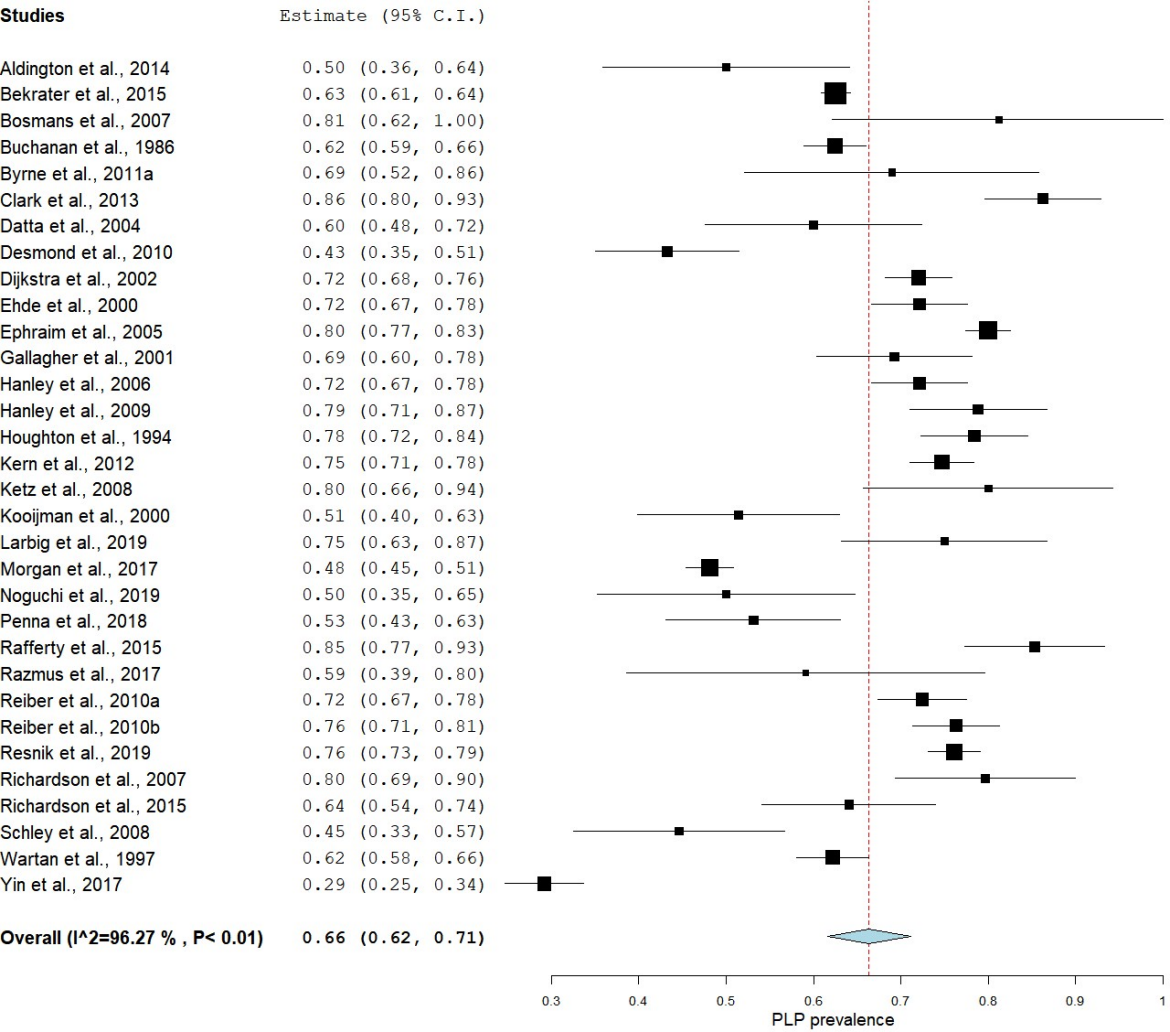
A subgroup analysis showing the pooled estimated prevalence of PLP in developed countries.

**Fig 7:**
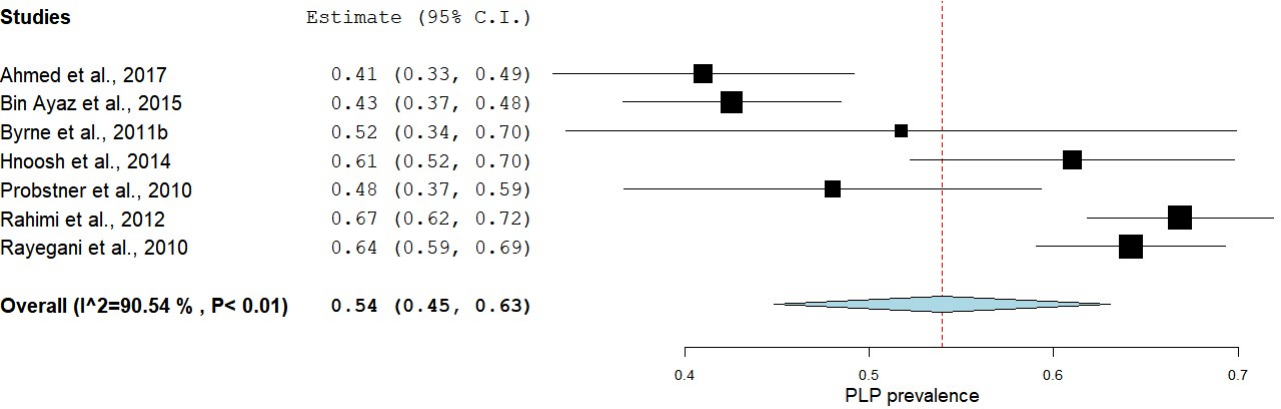
A subgroup analysis showing the pooled estimated prevalence of PLP in developing countries.

The subgroup analyses stratified by the developmental status of the countries in which the studies were conducted showed an estimated pooled prevalence of 66.55% [95% CI: 62.02–71.64] in developed countries and 53.98% [95% CI: 44.79–63.05] in developing countries (Figs 6 & 7). The Mann-Whitney U test showed a statistically significant difference between the prevalence estimates of these two meta-analyses [U = 57, p = 0.03].

### Risk factors for phantom limb pain

Twenty-five potential risk factors had been studied in 15 studies representing 4102 participants. Of these, 10 pre-amputation, three peri-operative and eight post-amputation risk factors had data to support their positive association with PLP, and six pre-amputation, four peri-operative and three post-amputation risk factors had not been found to be positively associated with PLP. The risk factors found to be positively associated with PLP and their measures of association are summarised in Table 2. Lower limb amputation was positively associated with PLP (moderate to strong association) in two studies representing a total of 1450 participants [12, 38]. Stump pain was consistently positively associated with PLP (weak to very strong association) in seven studies representing a total of 1254 participants [3, 4, 12, 13, 41, 46, 47]. Phantom sensations were consistently positively associated with PLP (strong to very strong association) in four studies representing a total of 1156 participants [12, 13, 42, 48]. Proximal site of amputation was positively associated with PLP (very strong association) in two studies representing a total of 604 participants [12, 49]. Diabetic cause of amputation was positively associated with PLP (moderate to strong association) in two studies representing a total of 580 participants [5, 12]. Persistent pre-amputation pain was positively associated with PLP in five studies representing a total of 881 participants (weak to very strong association) [3, 5, 9, 46, 50] but was not associated with PLP in two studies representing a total of 625 participants.

**Table 2:** The summary of the risk factors for PLP and their measures of association with PLP.

Author	Population	Outcome	Risk factor(s)	Measures of association	Strength of association
Ahmed et al., 2017	Cancer patients who had undergone limb amputations	PLP	Post-amputation depression	3.86 (1.75–8.53)^‡^	Strong
			Pre-amputation pain	2.83 (1.38–5.76)^‡^	Moderate
			Stump pain	31.2 (8.97–108.50)^‡^	Very strong
			Use of prosthesis	2.83 (1.19–4.76)^‡^	Moderate
			Sleep disturbance	21.43 (8.28–55.43)^‡^	Very strong
Buchanan et al., 1986	Amputees who were receiving routine prosthetic services	PLP	Age	0.12 (p<0.01)^¥^	Weak
Desmond et al., 2010	Members of the British Limbless Ex-Service Men’s Association	PLP	Stump pain	11.17 (p<0.01)^‡^	Very strong
Dijkstra et al., 2002	Amputees who were receiving routine prosthetic services	PLP	Diabetic cause of amputation	4 (p<0.001)^‡^	Strong
			Proximal site of amputation	1.60 (0.038)^‡^	Moderate
			Lower limb amputation	5.60 (p<0.001)^‡^	Strong
			Bilateral amputations	8.20 (p = 0.01)^‡^	Strong
			Stump pain	3.90 (p<0.001)^‡^	Strong
			Phantom sensations	19.50 (p<0.001)^‡^	Very strong
Ephraim et al., 2005	Amputees who had contacted the Amputee Coalition of America (ACA) between 1998 and 2000	PLP	Post-amputation depression	2 (1.3–3.1)^‡^	Moderate
			Lower limb amputation	2.50 (1.3–4.7)^‡^	Moderate
			2 or more comorbidities	2.70 (1.3–5.8)^‡^	Moderate
			Widow	2.70 (1.1–6.5)^‡^	Moderate
Gallagher et al., 2001	Amputees who were attending the Limb Fitting Clinic.	PLP	Proximal site of amputation	15.65 (p<0.001)^‡^	Very strong
			Traumatic cause of amputation	14.60 (p<0.002)^‡^	Very strong
			Sex (male)	3.76 (p<0.05)^‡^	Strong
			Other medical problems	5.93 (p<0.02)^‡^	Strong
			Lack of pre-amputation counselling	4.74 (p<0.03)^‡^	Strong
Hanley et al., 2009	Patients who had undergone upper-limb amputation 6 months or more before recruitment	PLP	Use of prosthesis	4.23 (p<0.05)^¶^	Moderate
Hanley et al., 2006	Patients who had undergone lower limb amputation	PLP	Pre-amputation pain	0.48 (p<0.01)^§^	Weak
			Stump pain	0.53 (p<0.0001)^§^	Weak
Kooijman et al., 2000	Amputees using upper limb prosthesis	PLP	Phantom sensations	11.30 (p = 0.001)^†^	Very strong
			Stump pain	1.90 (p = 0.015)^†^	Weak
Larbig et al., 2019	Patients who had undergone upper or lower limb amputations	PLP	Pre-amputation depression	2.05 (p<0.05)^§^	Moderate
			Pre-amputation pain	4.22 (p<0.01)^§^	Moderate
			Stump pain	3.90 (p<0.01)^§^	Moderate
Noguchi et al., 2019	Patients who had undergone upper or lower limb amputations	PLP	Diabetic cause of amputation	2.24 (p = 0.032)^‡^	Moderate
			Pre-amputation pain	6.36 (p = 0.024)^‡^	Strong
Razmus et al., 2017	Occupants of the nursing home, and clients of the Public Institute of Orthopaedic Equipment	PLP	Phantom sensations	4.94 (P<0.05)^§^	Strong
Richardson et al., 2007	Patients who had undergone amputation of the lower limb due to peripheral vascular disease.	PLP	Stump pain	7.03 (1.34–36.82)^‡^	Strong
			Increased ability to move the phantom limb.	8.31 (1.54–44.79)^‡^	Strong
			Praying/hoping	2.86 (1.68–13.18)^‡^	Moderate
			Catastrophizing	3.28 (1.71–14.91)^‡^	Strong
			Passive coping	4.60 (6.50–25.00)^‡^	Strong
Wartan et al., 1997	Traumatic amputees	PLP	Phantom sensations	107.30 (p<0.0001)^§^	Strong
Yin et al., 2017	Amputees who underwent limb amputations at a tertiary hospital	PLP	Pre-amputation pain	10.40 (p = 0.002)^‡^	Very strong
			Post-amputation epidural analgesia	4.90 (p = 0.008)^‡^	Strong

¥ _Point-biserial correlation analysis;_

_¶ Pearson’s univariate correlation test;_

_§ Chi-squared;_

_† Relative risk;_

‡ _Odds ratio._

The risk factors which were not found to be positively associated with PLP are summarised in Table 3. Sex, age and traumatic cause of amputation were the most commonly assessed of these proposed risk factors. Sex was consistently not associated with PLP in six studies representing a total of 1836 participants [3, 5, 13, 38, 51, 52]. Age was not associated with PLP in three studies representing a total of 1062 adult participants [5, 38, 52] but higher age was positively associated with PLP (weak association) in one study representing a total of 716 adult participants [53]. A traumatic cause of amputation was not associated with PLP in two studies representing a total of 958 participants [5, 38] but was positively associated with PLP (very strong association) in one study representing a total of 104 participants [54]. The meta-analysis of risk factors for PLP could not be conducted because of variations in methods of data collection and analysis across the included studies.

**Table 3:** The summary of factors not associated with increased risk for PLP and their measures of association with PLP.

Author	Population	Outcome	Risk factor(s)	Measures of association
Ahmed et al., 2017	Cancer patients who had undergone limb amputations	PLP	Sex	0.65 (0.31–1.40)^‡^
			smoking	1.40 (0.71–2.78)^‡^
			Regional Anaesthesia	0.99 (0.68–1.54)^‡^
			Post-amputation analgesia	1.41 (0.94–2.10)^‡^
			Perioperative gabapentin	0.75 (0.76–1.51)^‡^
			Radiotherapy	1.33 (0.66–2.66)^‡^
Dijkstra et al., 2002	Amputees who were receiving routine prosthetic services	PLP	Sex	— (p = 0.73)^‡^
			Prosthesis use (>8 hours per day)	— (p<0.13)^‡^
Ephraim et al., 2005	Amputees who had contacted the Amputee Coalition of America (ACA) between 1998 and 2000	PLP	Sex	1.4 (0.90–2.20)^‡^
			Age	1.1 (0.60–1.80)^‡^
			Traumatic cause of amputation	0.9 (0.50–1.70)^‡^
			Years since amputation	1.0 (0.60–1.90)^‡^
Gallagher et al., 2001	Amputees who were attending the Limb Fitting Clinic.	PLP	Post-amputation support	— (—)
Hanley et al., 2009	Patients who had undergone upper-limb amputation 6 months or more before recruitment	PLP	Age	3.78 (p = 0.83)^¶^
			Sex	0.78 (p = 0.99)^¶^
Kooijman et al., 2000	Amputees using upper limb prosthesis	PLP	Sex	— (p = 0.21)^†^
			Amputation of the dominant limb	— (p = 0.59)^†^
			Pre-amputation pain	— (p = 0.59)^†^
			Upper limb amputation	— (p = 0.08)^†^
			Prosthesis use (>8 hours per day)	— (p = 0.06)^†^
Noguchi et al., 2019	Patients who had undergone upper or lower limb amputations	PLP	Sex	0.78 (p = 0.73)^‡^
			Age	— (p = 0.65)^‡^
			Traumatic cause of amputation	2.941 (p = 0.22)^‡^
			Increased hospital-stay	— (p = 0.26)^‡^
Wartan et al., 1997	Traumatic amputees	PLP	Pre-amputation pain	10.6 (p<0.30)^§^

¥ _Point-biserial correlation analysis;_

_¶ Pearson’s univariate correlation test;_

_§ Chi-squared;_

_† Relative risk;_

‡ _Odds ratio;—missing figure._

## Discussion

According to our knowledge, this is the first systematic review to pool the literature on the prevalence and risk factors for PLP in people with limb amputations. The results of this study estimate that PLP affects 64% of people with amputations. Furthermore, this study identified that lower limb amputation, stump pain, phantom sensations, persistent pre-amputation pain, proximal site of amputation and diabetic cause of amputation are risk factors for PLP.

### Phantom limb pain prevalence

The current meta-analysis estimated that 64% of people with amputations report PLP. This estimate suggests that approximately 8169 of 12765 participants in this study reported PLP. Interestingly, dividing studies by risk of bias revealed no difference in estimated prevalence, despite the ‘low risk of bias’ subgroup’s meta-analysis having lower statistical heterogeneity. In addition, the results of the Egger’s regression test indicated that the asymmetry of the funnel plot (S5 File) was not significant (p = 0.64), thus failing to suggest the presence of publication bias. Altogether, these findings suggest that the included studies provide a reasonably stable estimate of the prevalence of PLP in the population of people with amputations. The prevalence of PLP appears to be high, supporting that health professionals should be aware of the risk of this complication and that pragmatic interventions for preventing or alleviating PLP are needed.

The meta-analysis that stratified the studies by country developmental status suggested that the prevalence of PLP was significantly lower in developing countries compared to developed countries [53.98% vs 66.55%; p = 0.03]. This discrepancy is surprising and might be an artefact of selection bias linked either to the lower recruitment success rates (57.9% - 68.4%) seen in most of the included studies conducted in developing countries [3, 34]. The strategy of recruiting participants from amongst patients receiving follow-up medical care may have contributed to underestimation of PLP prevalence if amputees with PLP without continuing medical care were excluded from samples (in developing countries), or overestimation if having PLP made amputees more likely to remain in medical care (in developed countries). This lack of clarity regarding recruitment strategies highlights the need to adapt recruitment strategies specifically to people with amputations in developing countries so that they can be accounted for in future studies.

The current literature suggests the standard of surgical care in developed countries differs significantly from that in developing countries. A study investigating the global burden and distribution of surgery revealed that approximately 80% more surgery-related complications and deaths occur in developing than in developed countries, despite accounting for only 26% of surgical procedures conducted globally [55]. These data may reflect the disparity in the standard of surgical care between developed and developing countries. Many healthcare facilities in developing countries, particularly in rural areas, have poor infrastructure and lack essential surgical equipment and skilled surgeons [56]. Urban areas may have a few skilled surgeons, yet the need for surgical care is typically greater in the rural parts of developing countries. As a result, surgical procedures are often conducted by less trained healthcare professionals under sub-standard conditions. Surgical care in developing countries therefore tends to be substandard than that in developed countries [56].

Another important consideration is that the prevalence estimates could have been influenced by the under-representation of only seven studies conducted in developing countries compared to 32 studies conducted in developed countries. Trauma or combat-related amputations are common in some regions in South America, Middle East, and West and Central Africa [57–59]. However, these regions are underrepresented in the body of studies identified by this review. In fact, we could not find any relevant study conducted in the continent of Africa. This highlights a concerning dearth of scientific research on PLP in these developing regions. Therefore, we recommend that further studies be focused in the burden of PLP in developing countries, specifically.

The included studies had varying risk of bias. However, the lack of statistically significant difference between the prevalence estimates from pooling of the studies with low risk of bias and pooling of the studies with moderate-high risk of bias suggests that the overall risk of bias in included studies had little impact on the prevalence of PLP. Nonetheless, the high risk of bias attributed to most studies for using an ambiguous definition of PLP (e.g pain felt in the limb after amputation) leaves the possibility that participants might have confused residual limb pain and PLP. We suspect that this might have resulted in an overestimation of the prevalence of PLP.

We found it interesting that the pooled prevalence estimate of PLP in this study was relatively high compared to that reported in the literature on people with congenitally absent limbs. The three studies available on people with congenitally absent limbs (not eligible for this review) reported a markedly low PLP prevalence of 0% (out of 27 participants), 5.7% (out of 88 participants) and 7% (out of 57 participants) [6, 13, 60]. Although a robust conclusion cannot be drawn from three small studies, these findings suggest that people with congenitally absent limbs may be less likely to experience PLP than those whose amputations were due to trauma or surgery [54]. Perhaps the peripheral nerves severed during amputation play an important role in the initiation of PLP after amputation [61]. In addition, the absence of pre-operative and peri-operative risk factors for PLP in this group might contribute to the low prevalence.

### Risk factors for phantom limb pain

Five studies showed that PLP was more likely to occur in people who reported a history of persistent pre-operative pain than in those who did not report having had persistent limb pain prior to their amputation. One physiological mechanism that has been proposed to explain the link between pre-amputation pain and PLP is central sensitisation—where persistent pre-operative pain contributes to the hyperexcitability of the nervous system and functional changes in the cortical areas involved in the generation of pain [62]. These changes may continue to upregulate peripheral input after limb amputation, thus promoting PLP that shares the characteristics with pre-amputation pain [63]. In fact, over 60% of the patients who experienced persistent pre-amputation pain reported similar characteristics of their PLP [64, 65]. This apparent relationship highlights the importance of addressing limb pain very early in patients who are at high risk of having their limbs amputated. The early management of pre-amputation pain using effective treatments such as pre-operative epidural analgesia (e.g. ketamine) and mirror therapy may reduce risk of developing PLP and improve physical and psychological outcomes often related to delayed or ineffective management of PLP [46, 66].

Two studies showed that PLP was more likely to occur after lower limb amputation than after upper limb amputation [38, 51]. The authors proposed that the use of a cosmetic prosthetic leg, rather than a prosthesis that provided sensory input was a likely contributor to pain in people with lower limb amputations since 70%-78.8% of cosmetic prosthetic leg users had PLP. Lack of proprioceptive feedback during the use of a prosthetic leg has been linked to poor motor control, possibly leading to stump irritation that may trigger PLP [2, 67]. This proposed link is partially supported by seven studies in this review which suggested that PLP was more likely to occur in people with stump pain than in those without stump pain [3, 4, 12, 13, 41, 44, 47]. Interestingly, Dietrich and colleagues investigated the effects of a leg prosthesis with somatosensory feedback on pain and lower limb function [68]. In that study, participants used prosthetic legs with pressure sensors that provided comfortable electrical feedback to the patient’s thigh whenever the prosthetic foot touched the ground. At the end of two weeks of training, the participants had improved function of the lower limb and reduced severity and frequency of PLP. Further, the patients reported greater satisfaction, longer walking distances and improved dynamic stability than prior to the training. These results suggest that people with lower limb amputations might benefit more from using a prosthetic leg with somatosensory feedback than from using a cosmetic prosthesis. However, the mechanisms by which prosthetic legs with somatosensory feedback reduce PLP are not clear. Therefore, it would be interesting to investigate the mechanisms by which somatosensory feedback from a prosthetic leg might influence PLP.

Four studies showed that PLP was more likely to occur in amputees with non-painful phantom sensations than in those without non-painful phantom sensations [13, 42, 48]. In these studies, 70%-100% of amputees with phantom sensations also had PLP. The co-occurrence of these post-amputation sensations suggest that they may share neural mechanisms with PLP [48]. An fMRI study by Andoh et al showed that inducing non-painful phantom sensations in people with amputations activated the somatosensory and premotor cortices contralateral to the amputated limb [69]. The activation of similar cortical areas has been recorded in patients with PLP upon induction of their PLP [70–74]. The similarities in cortical activation patterns might explain a link between PLP and non-painful phantom sensations.

Two studies showed that PLP was more likely to occur in people with proximal amputations than in those with distal amputations [12, 54]. These findings line up with a narrative review that reported an increase in the incidence of PLP with more proximal amputations [75]. Proximal amputations are associated with an increased risk of failure of wound healing, which may result in infection or stump pain [76]. However, the reasons why proximal amputations should be more likely to lead to PLP than distal amputations are not clear [77].

Another interesting finding was that not having pre-amputation counselling was positively associated with PLP (strong association) in a study representing a total of 104 participants [54]. This suggests that patients who receive counselling prior to their amputation maybe less likely to report PLP compared to those who do not receive counselling. We could not find any relevant study to explain this strong association. However, Gallagher et al [54] suggest that pre-amputation counselling may reduce the risk of developing PLP by addressing depression and anxiety prior to limb amputation [78]. Another consideration is that pre-amputation counselling aimed at managing patients’ expectations about pain post-operatively (e.g. prognosis) and equipping them with adaptive coping strategies may reduce the risk of PLP by preventing the onset of post-amputation depression that is sometimes triggered by the feeling of helplessness from the overwhelming new reality of life after limb amputation [38]. No other study has specifically identified not having pre-operative counselling as a predictor for PLP after limb amputation. Therefore, further studies are required to build on the existing literature.

### Limitations

The sample in this systematic review was skewed towards males, in that 9814 (77.04%) of the 12738 participants were male. Therefore, the results might not hold for females. We could not perform a subgroup analysis by sex because we did not have individual patient data, nor was analysis by sex an objective identified in the protocol. However, the data on risk factors provide no support for sex influencing the likelihood of PLP after amputation. It was not possible to conduct a meta-analysis on the risk factors for PLP because the included studies used varying methodological approaches and measures of association. None of the included studies used an outcome measure that has been validated for assessing PLP. In fact, we are not aware of any instrument that has been validated for assessing PLP. Such a standardised tool for assessing PLP would be useful to provide us with reliable data. Most studies in this review had moderate-high risk of bias. There is a clear need for high-quality studies to raise the credibility of future meta-analyses. Finally, the search strategy for this study was designed specifically to identify prevalence studies. Therefore, although we did conduct an exploratory search for additional studies of risk factors for PLP, there is a possibility that we could have missed some studies that investigated risk factors for PLP if they did not also estimate PLP prevalence. In consideration of this possibility, the review of risk factors was classified as an exploratory analysis. Thirteen out of 15 included studies determined association between identified risk factors and PLP using a retrospective cross-sectional study design. This study design (compared to a cohort design) is prone to recall bias, resulting from the patient’s inability to clearly recall their exposure to a risk factor prior to developing PLP. Further studies using a cohort design are necessary to provide robust data on risk factors for PLP. It is important to note that studies conducted in developing countries are underrepresented in our meta-analyses. Therefore, the results on PLP prevalence in developing countries should be interpreted with caution. The results of this systematic review were derived from studies conducted mostly in Europe, North America and Asia. To the best of our knowledge, no study has been conducted in Africa, and research in this area is necessary to inform us about the prevalence and risk factors for PLP in the African population.

## Conclusions

This systematic review and meta-analysis estimates that six of every 10 people with an amputation report PLP–a high and important prevalence of PLP. Health care professionals ought to be aware of the high rates of PLP and implement strategies to reduce PLP by addressing known risk factors, specifically those identified by the current study. Stump pain and post-amputation depression are all known and modifiable risk factors that are consistently positively associated with PLP. Awareness of these risk factors may motivate health care professionals to address them early in treatment to prevent the onset of PLP in people with amputations.

## Supporting information

S1 File PRISMA checklist. (DOC)

S2 File Customised search strategy. (DOCX)

S3 File Risk of bias assessment checklist for prevalence studies. (DOCX)

S4 File Summary of the risk of bias assessment results. (DOCX)

S5 File Funnel plot assessing publication bias in a meta-analysis of 39 included studies. (JPG)
