# Supplementary material for: The prevalence and risk factors for phantom limb pain in people with amputations: A systematic review and meta-analysis
Source: PLoS One. 2020 Oct 14;15(10):e0240431. doi: 10.1371/journal.pone.0240431 (PMC7556495; doi:10.1371/journal.pone.0240431)
Supplement: S2 File — (DOCX) [file pone.0240431.s002.docx]

S2 File: Customised Search strategy

1. Amputation [MeSH] OR Amputation, Traumatic [MeSH] OR Amputation Stumps [MeSH] OR Amputee OR amputees OR amputation OR limb deficiency OR limb loss.
2. Phantom Limb [MeSH] OR Phantom limb OR phantom pain OR phantom sensations OR phantom sensation OR residual limb pain
3. Epidemiology [MeSH] OR Epidemiology [Subheading] OR Prevalence [MeSH] OR Risk Factors [MeSH]
4. associated OR association OR burden OR case-control OR cohort OR correlation OR correlates OR cross-sectional OR determinant OR epidemiology OR epidemiological OR epidemiologic OR frequency OR incidence OR interview OR likelihood ratio OR observational OR occur OR occurrence OR odds ratios OR predict OR predictor OR prediction OR present OR presentation OR prevalence OR prevalent OR probability OR prognosis OR prognostic OR proportion OR prospective OR questionnaire OR questionnaires OR rate OR retrospective OR risk OR risks OR self-report OR statistic OR surveillance OR survey OR surveys
5. 1 AND 2 AND 3 AND 4
