# Supplementary material for: The prevalence and risk factors for phantom limb pain in people with amputations: A systematic review and meta-analysis
Source: PLoS One. 2020 Oct 14;15(10):e0240431. doi: 10.1371/journal.pone.0240431 (PMC7556495; doi:10.1371/journal.pone.0240431)
Supplement: S3 File — (DOCX) [file pone.0240431.s003.docx]

S3 File: Risk of bias assessment checklist for prevalence studies (Hoy et al [1])

| **Name of author(s):**  **Study title:**  **Year of study publication:** | | |
| --- | --- | --- |
| **Risk of bias items** | **Risk of bias levels** | **Point scored** |
| 1. Was the study’s target population a close representation of the national population in relation to relevant variables (e.g. number of limb amputations, participant’s sex and age)? | **Yes (LOW RISK)**: The study’s target population was a close representation of the national population in relation to relevant variables. | 0 |
|  | **No (HIGH RISK)**: The study’s target population was clearly NOT representative of the national population in relation to relevant variables. | 1 |
| 1. Was the sampling frame a true or close representation of the target population in relation to relevant variables (e.g. number of limb amputations, participant’s sex and age)? | **Yes** **(LOW RISK)**: The sampling frame was a true or close representation of the target population in relation to relevant variables. | 0 |
|  | **No (HIGH RISK)**: The sampling frame was NOT a true or close representation of the target population in relation to relevant variables. | 1 |
| 1. Was some form of random selection used to select the sample (e.g. simple random sampling, stratified random sampling, cluster sampling, systematic sampling), OR, was a census undertaken? | **Yes (LOW RISK)**: A census was undertaken, OR, some form of random selection was used to select the sample. | 0 |
|  | **No (HIGH RISK)**: A census was NOT undertaken, AND some form of random selection was NOT used to select the sample. | 1 |
| 1. Was the likelihood of non-response bias minimal? | **Yes (LOW RISK)**: The response rate for the study was ≥75%, OR, an analysis was performed that showed no significant difference in relevant demographic characteristics between responders and non- responders. | 0 |
|  | **No (HIGH RISK)**: The response rate was <75%, and if any analysis comparing responders and non-responders was done, it showed a significant difference in relevant demographic characteristics between responders and non-responders | 1 |
| 1. Were data collected directly from the study participants (as opposed to a proxy)? | **Yes (LOW RISK)**: All data were collected directly from the participants. | 0 |
|  | **No (HIGH RISK)**: In some instances, data were collected from a proxy, OR, it was unclear whether data were collected directly from participants. | 1 |
| 1. Was a clear and acceptable case definition used in the study (e.g painful sensation(s) felt in the amputated limb)? | **Yes (LOW RISK)**: An acceptable case definition was used. | 0 |
|  | **No (HIGH RISK)**: An acceptable case definition was NOT used. | 1 |
| 1. Was the study instrument that measured the outcome of interest (e.g. prevalence of PLP) shown to have reliability and validity (e.g. test-re-test, piloting, validation in a previous study)? | **Yes (LOW RISK)**: The study instrument had been shown to have reliability and validity. | 0 |
|  | **No (HIGH RISK)**: The study instrument had NOT been shown to have reliability or validity. | 1 |
| 1. Was the same mode of data collection used for all participants? | **Yes (LOW RISK)**: The same mode of data collection was used for all participants. | 0 |
|  | **No (HIGH RISK)**: The same mode of data collection was NOT used for all participants, OR, it was unclear whether the same mode of data collection was used for all participants | 1 |
| 1. Was the length of the shortest prevalence period for the outcome of interest appropriate | **Yes (LOW RISK)**: The point prevalence for the outcome of interest was reported, OR, the period prevalence on the outcome of interest was reported ≤ 1 year after limb loss. | 0 |
|  | **No (HIGH RISK)**: The point prevalence for the outcome of interest was NOT reported, AND, the period prevalence on the outcome of interest was reported > 1 year after limb loss. | 1 |
| 1. Were the numerator(s) and denominator(s) for the outcome of interest (e.g. the prevalence of PLP) appropriate? | **Yes (LOW RISK)**: The paper presented appropriate numerator(s) AND denominator(s) for the outcome of interest. | 0 |
|  | **No (HIGH RISK)**: The paper did NOT present numerator(s) AND denominator(s) for the outcome of interest, OR, the paper did present numerator(s) AND denominator(s) for the outcome of interest but one or more of these were inappropriate. | 1 |
| Summary on the overall risk of study bias | **Low risk** | **0-3** |
|  | **Moderate risk** | **4-6** |
|  | **High risk** | **7-10** |

1. Hoy, D., et al., *Assessing risk of bias in prevalence studies: modification of an existing tool and evidence of interrater agreement.* Journal of clinical epidemiology, 2012. **65**(9): p. 934-939.
