# Supplementary material for: The prevalence and risk factors for phantom limb pain in people with amputations: A systematic review and meta-analysis
Source: PLoS One. 2020 Oct 14;15(10):e0240431. doi: 10.1371/journal.pone.0240431 (PMC7556495; doi:10.1371/journal.pone.0240431)
Supplement: S4 File — (DOCX) [file pone.0240431.s004.docx]

S4 File: Summary of the risk of bias assessment results.

| Authors | Was the  Study’s target  population a close  representation  of the national  population? | Was the  sampling frame  a true or close  representation  of the target  population? | Was some  form of  random  selection used  to select the  sample, OR,  was a census  undertaken? | Was the  likelihood of  non-response  bias minimal? | Were data  collected  directly from  the participants? | Was an  acceptable case  definition used  in the study? | Was the study  instrument  shown to have  reliability and  validity? | Was the same  mode of data  collection used  for all  participants? | Was the length  of the shortest  prevalence  period for the  parameter of interest  appropriate? | Were the  Numerator and  denominator for the parameter of  interest appropriate? | Overall risk of bias |
| --- | --- | --- | --- | --- | --- | --- | --- | --- | --- | --- | --- |
| Ahmed et al., 2017 | High | Low | High | High | High | High | High | Low | Low | Low | Moderate |
| Aldington et al., 2014 | High | Low | Low | High | Low | Low | High | Low | Low | Low | Low |
| Bekrater et al., 2015 | Low | Low | High | High | Low | Low | High | High | Low | Low | Moderate |
| Bin Ayaz et al., 2015 | High | High | High | High | High | Low | High | High | High | Low | High |
| Bosmans et al., 2007 | High | Low | Low | High | Low | High | High | Low | Low | Low | Moderate |
| Buchanan et al., 1986 | High | High | High | High | Low | High | High | Low | High | Low | High |
| Byrne et al., 2011^a^ | High | High | Low | High | Low | High | High | High | High | Low | High |
| Byrne et al., 2011^b^ | High | High | Low | High | Low | High | High | High | High | Low | High |
| Clark et al., 2013 | High | Low | High | High | High | High | High | High | High | Low | High |
| Datta et al., 2004 | High | Low | High | Low | High | High | High | Low | High | Low | Moderate |
| Desmond et al., 2010 | High | Low | High | High | High | Low | High | Low | Low | Low | Moderate |
| Dijkstra et al., 2002 | High | Low | High | High | High | Low | High | Low | High | Low | Moderate |
| Ehde et al., 2000 | High | Low | Low | High | High | Low | High | Low | High | Low | Moderate |
| Ephraim et al., 2005 | Low | Low | Low | High | High | Low | High | Low | High | Low | Moderate |
| Gallagher et al., 2001 | High | High | High | High | High | High | High | Low | High | Low | High |
| Hanley et al., 2006 | High | Low | Low | High | High | Low | High | High | Low | Low | Moderate |
| Hanley et al., 2009 | High | High | Low | High | High | High | High | Low | High | Low | Moderate |
| Houghton et al., 1994 | High | Low | High | High | High | High | High | Low | High | Low | High |
| Hnoosh et al., 2014 | Low | High | Low | High | Low | Low | High | Low | Low | Low | Low |
| Kern et al., 2012 | Low | Low | Low | High | High | High | High | Low | High | Low | Moderate |
| Ketz et al., 2008 | High | Low | High | High | Low | Low | High | Low | High | Low | Moderate |
| Kooijman et al., 2000^a^ | High | Low | High | Low | High | High | High | Low | High | Low | Moderate |
| Kooijman et al., 2000^b^ | High | Low | High | Low | High | High | High | Low | High | Low | Moderate |
| Larbig et al., 2019 | High | High | High | High | Low | High | High | Low | Low | Low | Moderate |
| Morgan et al., 2017 | Low | Low | High | Low | High | High | High | Low | Low | Low | Moderate |
| Noguchi et al., 2019 | High | High | High | High | Low | Low | High | Low | Low | Low | Moderate |
| Penna et al., 2019 | High | High | Low | High | High | High | High | Low | High | Low | High |
| Probstner et al., 2010 | High | High | High | High | Low | High | High | High | High | Low | High |
| Rafferty et al., 2015 | High | High | High | Low | Low | High | High | Low | Low | Low | Moderate |
| Rahimi et al., 2012 | Low | Low | High | Low | Low | High | High | Low | Low | Low | Low |
| Rayegani et al., 2010 | High | Low | High | Low | Low | Low | High | Low | Low | Low | Low |
| Razmus et al., 2017 | High | High | High | High | Low | High | High | Low | High | Low | High |
| Reiber et al., 2010^a^ | High | low | High | High | High | High | High | High | High | Low | High |
| Reiber et al., 2010^b^ | High | low | High | High | High | High | High | High | High | Low | High |
| Resnik et al., 2019 | Low | Low | High | High | Low | High | High | Low | High | Low | Moderate |
| Richardson et al., 2007 | High | Low | High | Low | Low | High | High | Low | High | Low | Moderate |
| Richardson et al., 2015 | High | Low | High | High | Low | High | High | Low | Low | Low | Moderate |
| Schley et al., 2008 | High | Low | High | High | High | High | High | High | High | Low | High |
| Wartan et al., 1997 | High | Low | Low | Low | High | High | High | Low | High | Low | Moderate |
| Yin et al., 2017 | High | Low | Low | High | High | High | High | Low | High | Low | Moderate |
