# Supplementary figures and images for: The prevalence and risk factors for phantom limb pain in people with amputations: A systematic review and meta-analysis
Source: PLoS One. 2020 Oct 14;15(10):e0240431. doi: 10.1371/journal.pone.0240431 (PMC7556495; doi:10.1371/journal.pone.0240431)

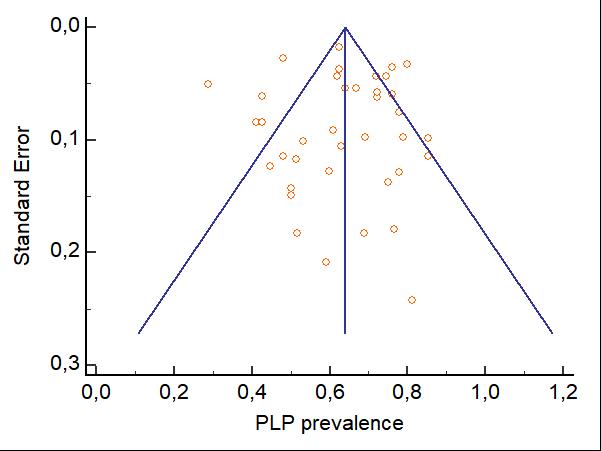

Supplement: S5 File — (JPG) [file pone.0240431.s005.jpg]
